# Patient-specific and provider-specific predictors of urgent care referral to dermatology: A retrospective cohort and survey study

**DOI:** 10.1016/j.jdin.2026.04.008

**Published:** 2026-04-16

**Authors:** Amanda Rodriguez Orengo, Alison Moynihan, Christopher T. Richardson, Clayton Green, Julie Ryan Wolf

**Affiliations:** a University of Rochester School of Medicine and Dentistry, Rochester, New York; b Department of Dermatology, University of Rochester Medical Center, Rochester, New York

**Keywords:** access, consults, demographics, dermatology, referrals, teledermatology, urgent care

## Abstract

**Background:**

Some patients are referred unnecessarily from urgent care (UC) to dermatology, while others who need additional care are not referred or do not complete the referral, highlighting the need for improved triage of skin concerns at UC centers.

**Objectives:**

To identify factors that predict UC referral to dermatology and to better understand UC perspectives on improving urgent access for skin concerns.

**Methods:**

This study includes a retrospective nominal logistic regression cohort analysis of 6988 skin-related UC encounters to identify predictors of referral to dermatology and a survey of UC perspectives on managing dermatologic conditions and improving dermatology access.

**Results:**

Non-White patients and those evaluated at UC sites closer to dermatology had higher odds of referral. Uninsured patients and those requiring procedures or ongoing follow-up were also more likely to be referred. Dermatology education for UC providers, teledermatology, and urgent-access clinics were favored to improve dermatologic care access.

**Limitations:**

Limitations include single-system design, unmeasured provider factors, limited disease severity assessment, and potential survey response bias.

**Conclusion:**

Several patient-related factors and UC proximity to dermatology influence UC referral to dermatology. Dermatology training for nondermatologists, teledermatology services to support UC, and dermatology-specific urgent-access clinics may improve patient triage and reduce unnecessary referrals.

**Capsule Summary**

• This study highlights patient-related (race, ethnicity, insurance, location, and diagnosis) and physician-related factors (diagnosis and treatment uncertainty) that affect referral to dermatology from urgent care.
• Urgent care providers support continuing education, teledermatology, and urgent access clinics as ways to improve urgent access to dermatologic care.

## Introduction

Referral decisions carry significant implications for patient outcomes and health care costs. While many referrals expedite appropriate diagnosis and treatment, some result in unnecessary testing and procedures.^1^^,^^2^ Specialists often use more health care resources, leading to increased costs when referrals are unnecessary.^3^ Conversely, referral requirement by some insurance plans impedes specialty care access. Our previous findings revealed that 17.5% of urgent care (UC) referrals to dermatology were unnecessary according to a dermatologist expert panel.^4^ Furthermore, 20% of referred patients received suboptimal treatment at UC, raising concern that those not referred might also have received inadequate care.^4^ Improved referral triage is crucial for reducing unnecessary referrals and optimizing dermatologic care in UC.

Both provider-related and patient-related factors can contribute to referral decisions. Patient-related factors shown to influence referrals include the presenting problem, comorbidities, patient demographics (eg, age, gender, race/ethnicity, insurance status, income), and the patient’s desire for referral.^5^, ^6^, ^7^, ^8^ Provider-related factors include provider characteristics (eg, age, specialty, years of experience), workload, and diagnostic uncertainty.^9^, ^10^, ^11^ Additionally, referring physician location also affects referrals with rural physicians having lower referral rates than their urban counterparts.^12^^,^^13^ Effective approaches proven to enhance the outpatient specialty referral process include referral checklists and educational activities with both consulting and referring providers.^14^ Both teledermatology and urgent dermatology clinics assist with dermatology triage and access.^15^^,^^16^

In this study, we aim to expand on our prior findings showing a flawed UC triage process by identifying underlying factors that predict referral to dermatology and eliciting UC provider perspectives on improving urgent access for skin concerns. Understanding why UC providers refer to dermatology is important to guide future improvements to the process.

## Methods

### Study design, setting, and participants

This study includes a retrospective cohort analysis and provider survey approved by the University of Rochester (UR) Research Subjects Review Board (STUDY00009636). We analyzed all skin-related encounters (*n* = 6988; 785 referred, 6203 nonreferred) across 8 UR-affiliated UC sites from September 1, 2022 to March 31, 2024, using electronic health record data extracted by UR Clinical & Translational Science Institute. Referrals were defined as provider-initiated orders for outpatient dermatology evaluation. Manual chart review determined primary care provider (PCP) status for a subset of encounters (*n* = 1296; 785 referred, 511 nonreferred).

### Retrospective study

We evaluated patient-level predictors of dermatology referral, including age, sex, race, ethnicity, interpreter need, insurance type, UC location, diagnosis, PCP status, and Social Deprivation Index (SDI). SDI measures domains such as income, education, employment, housing, and transportation by zip code.^17^ SDI scores were categorized as “high deprivation” if in the top quartile (SDI ≥62) and “low deprivation” otherwise. We used SDI ≥62 for “high deprivation” for all analyses to maintain generalizability despite the higher top quartile value for SDI (≥74) in the smaller 1296 encounter subset. Driving distance from each UC site to the primary UR dermatology clinic was calculated to assess whether proximity influenced referrals. Similar diagnoses were grouped and nonspecific diagnostic codes (International Classification of Diseases, 10th Revision: R22.9, R23.8, R23.4) were categorized as “unspecified skin condition.” Only diagnoses with >10 encounters were included in analyses. Referrals were considered complete if the patient attended a dermatology appointment. Wait time was calculated as days between date of referral and date of scheduled dermatology appointment.

### UC provider survey

A survey evaluating UC provider perspectives on dermatologic care was distributed via institutional email, using REDCap, to all UR UC providers (*n* = 60). The survey assessed: (1) frequency of dermatologic cases encountered in UC; (2) frequency and reasons for referrals to dermatology; (3) confidence levels in diagnosing and treating dermatologic conditions; (4) preferred learning formats for dermatology education; and (5) attitudes toward potential solutions to improve dermatologic care access, including synchronous and asynchronous teledermatology consultations. Synchronous consultations were defined as phone-based consultations made during the UC visit. Asynchronous consultations involved sending clinical information to a provider with a response expected within 24 to 48 hours. Three board-certified dermatologists created the survey and confirmed content relevance and clarity with a UC provider prior to distribution.

### Data handling and statistical analysis

Analyses were performed at a 5% significance level using JMP Pro 17 or Python. Descriptive statistics summarized referred and nonreferred groups and survey responses. The primary analyses (ie, Pearson χ^2^ and analysis of variance tests) identified variables associated with referral (Table I) which were then included in a nominal logistic regression (multivariable) model to determine potential predictors of referral. Independent variables included race, age, ethnicity, interpreter need, insurance type, SDI, encounter location, and diagnosis. The dependent variable was referral status (ie, referred vs not referred). Secondary analyses (ie, χ^2^ tests and nominal logistic regression) assessed association of demographic variables (ie, race, ethnicity, SDI, insurance) with PCP status using a smaller subset of encounters (*n* = 1296). Nominal logistic regression determined if PCP status was associated with referrals. Due to the smaller sample size, encounter location and diagnosis were not included.

**Table I:** Comparison of referred and nonreferred patient demographic factors and encounter locations

	Referred, *n* = 785	Nonreferred, *n* = 6203	*P* value
Age (mean ± SD)	39.7 ± 19.9	43.6 ± 23.5	<.0001
Sex (*n*, %)			.5072
Female	415 (52.9)	3357 (54.1)	
Male	370 (47.1)	2846 (45.9)	
Race (*n*, %)			<.0001
White	531 (67.6)	5181 (83.5)	
Not White	240 (30.6)	975 (15.7)	
Unknown/refused	14 (1.8)	47 (0.8)	
Ethnicity (*n*, %)			.0007
Hispanic/Latino	52 (6.6)	307 (5.0)	
Not Hispanic/Latino	5649 (87.0)	683 (91.0)	
Unknown/refused	50 (6.4)	247 (4.0)	
Interpreter needed (*n*, %)			.0001
Yes	29 (3.7)	106 (1.7)	
No	756 (96.3)	6098 (98.3)	
SDI			<.0001
High SDI (≥62)	261 (33.2)	1487 (24.0)	
Low SDI (<62)	524 (66.8)	4717 (76.0)	
Insurance (*n*, %)			<.0001
Private	368 (46.9)	2866 (46.2)	
Medicaid	258 (32.9)	1672 (27.0)	
Medicare	131 (16.7)	1564 (25.2)	
Uninsured	23 (2.9)	46 (0.7)	
Veteran’s	5 (0.6)	56 (0.9)	
Urgent care (*n*, %)			<.0001
Location 1 (4.1 mi)	181 (23.1)	939 (15.1)	
Location 2 (5.3 mi)	143 (18.2)	865 (14.0)	
Location 3 (9.2 mi)	166 (21.1)	981 (15.8)	
Location 4 (10.4 mi)	141 (18.0)	948 (15.3)	
Location 5 (12.8 mi)	63 (8.0)	529 (8.5)	
Location 6 (14.1 mi)	63 (8.0)	795 (12.8)	
Location 7 (29.6 mi)	17 (2.2)	526 (8.5)	
Location 8 (64.5 mi)	11 (1.4)	621 (10.0)	

*SD*, Standard deviation; *SDI*, Social Deprivation Index.

## Results

### Study population

A total of 6988 patients were evaluated for dermatologic concerns across 8 UC sites, of whom 785 (11.2%) were referred to dermatology. Patients were offered the earliest available appointments and could choose among 3 UR dermatology locations. Only 47.9% of referred patients completed their referral, with a mean appointment wait time of 39.2 ± 50.8 days. Wait times did not differ by diagnosis (*P* = .6559). Notably, 23.7% of referred patients (*n* = 186) did not receive any treatment during their UC visit and nearly half of these patients (45.7%, *n* = 85) never completed their referral, suggesting that some patients never received treatment from UC or dermatology. The most common reason for incomplete referral was “no patient response” to scheduling attempts (38.5%, *n* = 165).

We observed differences across several demographic and clinical characteristics between referred and nonreferred patients (Table I). Referred patients were younger and more likely to be non-White, Hispanic/Latino, and require an interpreter. They were also more likely to reside in areas of higher socioeconomic deprivation, have Medicaid, or be uninsured. UC sites closer to dermatology had higher referral rates (Table I).

### Predictors of referral

**Fig 1:**
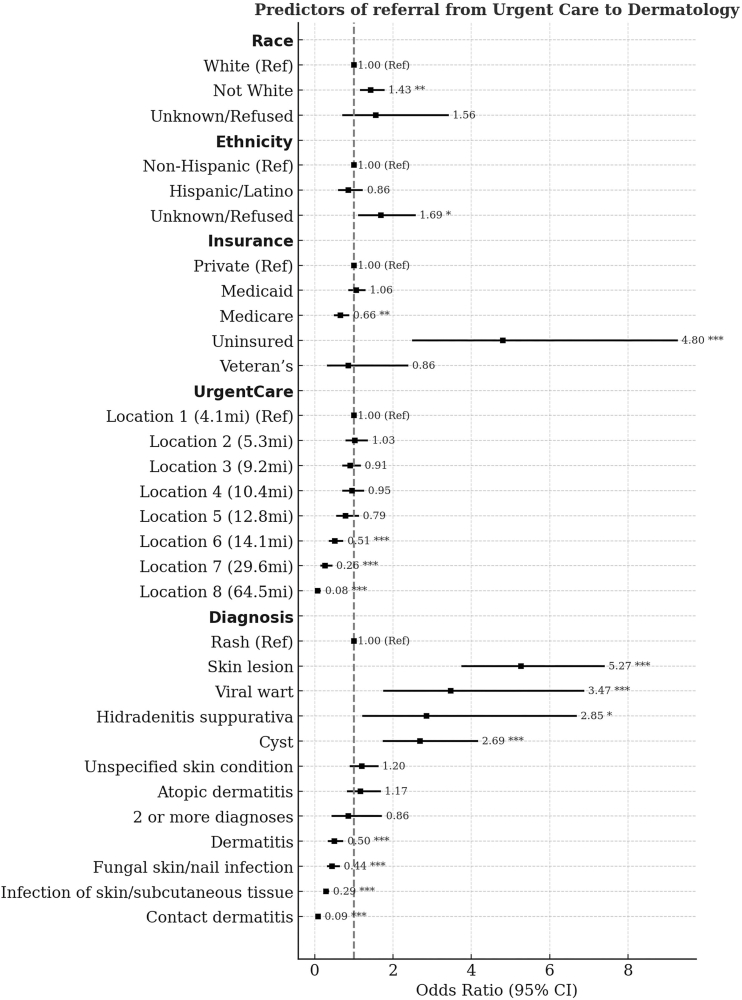
Predictors of referral from urgent care to dermatology. Adjusted odds ratios (ORs) and 95% confidence intervals (CIs) for predictors of dermatology referrals among urgent care (UC) encounters are displayed, grouped by race, ethnicity, insurance status, UC location, and diagnosis. Reference categories are noted as (Ref). *Asterisks* denote statistically significant predictors (∗*P* < .05, ∗∗*P* < .01, ∗∗∗*P* < .001).

Nominal logistic regression showed race, ethnicity, insurance status, UC location, and diagnosis as significant predictors of UC referral to dermatology, but not patient age, interpreter need, or SDI (Fig 1). Non-White patients (odds ratio [OR] = 1.43, 95% confidence interval [CI]: 1.16-1.78; *P* = .001) and patients who declined to report their ethnicity (OR = 1.69, 95% CI: 1.11-2.58; *P* = .014) had higher odds of referral compared to White patients. These results suggest that UC providers may be less comfortable managing skin conditions in patients with skin of color and/or from racially and ethnically diverse backgrounds.

Compared to the nearest UC clinic, patients seen at more distant sites had lower odds of referral (Fig 1). Locations 6, 7, and 8 had particularly reduced odds, with ORs of 0.51 (95% CI: 0.36-0.73; *P* = .0002), 0.26 (95% CI: 0.15-0.45; *P* < .0001), and 0.08 (95% CI: 0.04-0.16; *P* < .0001), respectively.

Compared to rash, higher odds of referral were observed for diagnoses requiring procedures or ongoing follow-up such as skin lesion (OR = 5.27, 95% CI: 3.75-7.40; *P* < .0001), viral wart (OR = 3.47, 95% CI: 1.75-6.88; *P* = .0004), hidradenitis suppurativa (OR = 2.85, 95% CI: 1.22-6.69; *P* = .016), and cyst (OR = 2.69, 95% CI: 1.74-4.17; *P* < .0001). Lower odds of referral were found for diagnoses that commonly require a short course of treatment like dermatitis (OR = 0.50, 95% CI: 0.34-0.73; *P* = .0004), fungal skin or nail infections (OR = 0.44, 95% CI: 0.31-0.64; *P* < .0001), infections of the skin and subcutaneous tissue (OR = 0.29, 95% CI: 0.22-0.37; *P* < .0001), and contact dermatitis (OR = 0.09, 95% CI: 0.06-0.15; *P* < .0001).

Insurance status was also a strong predictor. Uninsured patients had higher odds of referral compared to those with private insurance (OR = 4.80, 95% CI: 2.49-9.27; *P* < .0001), while Medicare coverage was associated with lower odds (OR = 0.66, 95% CI: 0.49-0.88; *P* = .005). However, uninsured patients were significantly less likely to complete their referral than insured patients (21.7% vs 48.7%; *P* = .011). Insurance status is likely not considered at the time of referral since our dermatology clinics do accept patients without insurance, but the potential for significant out-of-pocket costs likely deters many uninsured patients from completing the referral.

### PCP status subset

**Fig 2:**
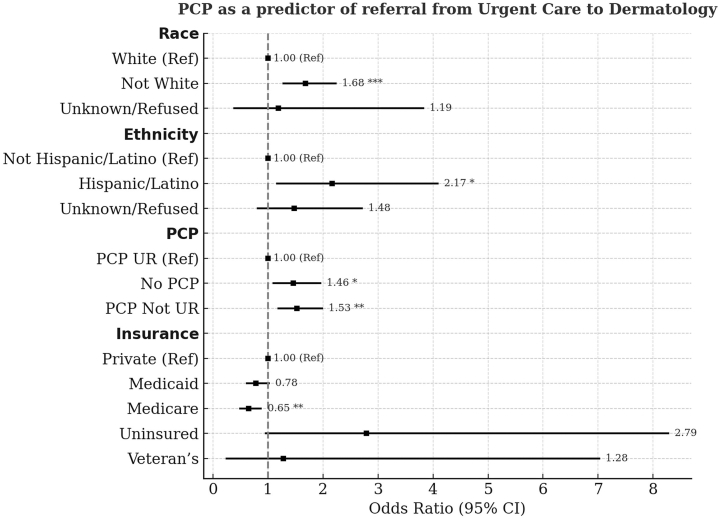
PCP as a predictor of referral from urgent care to dermatology. Adjusted odds ratios (ORs) and 95% confidence intervals (CIs) for predictors of dermatology referrals among urgent care (UC) encounters are displayed, grouped by race, ethnicity, insurance status, and PCP status. Reference categories are noted as (Ref). *Asterisks* denote statistically significant predictors (∗*P* < .05, ∗∗*P* < .01, ∗∗∗*P* < .001). *PCP*, Primary care provider.

One possible explanation for the higher referral rate among uninsured patients could be the lack of a PCP, which we explored in a subset analysis of 785 referred and 511 nonreferred patients. Approximately 76.8% of patients in this subset had a PCP documented in the electronic health record. Nominal logistic regression showed that patients with high SDI (OR = 0.63; 95% CI: 0.47-0.84; *P* = .0016) and those who were uninsured (OR = 0.17; 95% CI: 0.07-0.39; *P* < .0001) had lower odds of having a PCP as compared to those with private insurance, while Medicare patients had higher odds (OR = 2.12; 95% CI: 1.41-3.18; *P* = .0003). Overall, patients with a UR-affiliated PCP were less likely to be referred compared to those with a non-UR–affiliated PCP or no PCP (45.4% vs 34.9% vs 33.6%; *P* = .0002). Race, ethnicity, insurance, and PCP status were confirmed as referral predictors by nominal logistic regression (Fig 2).

### UC survey

Thirty-one UC providers completed a survey (31/60, 51.7% response rate) regarding management of dermatologic conditions and improving urgent access to dermatologic care (Table II). Most (71.0%) reported managing 2 to 5 skin concerns daily, highlighting the burden of dermatologic issues in UC. Dermatology referrals were made “some of the time” by 64.5% of respondents and “rarely” by 29.0%. The top 3 reasons for referring patients were “specialized procedure needed” (74.2%), “severe skin condition” (67.7%), and “chronic condition needing ongoing care” (60.0%), consistent with findings from our retrospective analysis. UC provider confidence in managing dermatologic conditions was moderate overall, with 60.0% reporting they felt confident “some of the time.”

**Table II:** Dermatologic care and urgent access survey responses from 31 urgent care providers

Survey question	Survey response	*n* = 31	%
1. Approximately how many patients with dermatologic conditions do you see on an average day?	0-1	1	3.2
	2-5	22	71.0
	6-10	7	22.6
	11+	1	3.2
2. How often do you refer patients with dermatologic conditions to dermatology?	Never	0	0.0
	Rarely	9	29.0
	Some of the time	20	64.5
	Most of the time	2	6.5
	Always	0	0.0
3. How often do you feel confident in your diagnosis and treatment of dermatologic conditions?	Never	0	0.0
	Rarely	1	3.3
	Some of the time	18	60.0
	Most of the time	11	36.7
	Always	0	0.0
4. What prompts you to refer a patient to dermatology? (pick top 3)	Specialized procedure needed	23	74.2
	Severe skin condition	21	67.7
	Chronic condition needing ongoing care	18	60.0
	Unsure about diagnosis	18	58.1
	Unsure about treatment approach	7	22.6
	Patient requested referral	3	9.7
	Other (please specify): No PCP for follow-up	1	3.2
5. Which educational formats would you take advantage of to increase dermatology proficiency? (choose all that apply)	Lecture series	22	71.0
	Self-study modules	22	71.0
	Virtual dermatology shadowing	13	41.9
	In-person dermatology shadowing	11	35.5
	None of the above	2	6.5
6. There needs to be more access to dermatologists for urgent skin issues.	Agree	28	90.3
	Neutral	3	9.7
	Disagree	0	0.0
7. An urgent dermatology clinic would enhance patient access to dermatology services.	Agree	30	96.8
	Neutral	1	3.2
	Disagree	0	0.0
8. I would use synchronous dermatology consultation by phone to aid during an urgent care visit.	Agree	26	83.9
	Neutral	4	12.9
	Disagree	1	3.2
9. I would use asynchronous store-and-forward teledermatology consult following an urgent care visit.	Agree	21	67.7
	Neutral	7	22.6
	Disagree	3	9.7
10. Would you prefer synchronous or asynchronous eConsult services?	Synchronous	22	71.0
	Asynchronous	9	29.0
	Neither	0	0.0
11. If you had access to eConsult services, would this change your likelihood of sending in-person referrals?	More likely to send referrals	3	9.7
	About the same	8	25.8
	Less likely to send referrals	20	64.5

*PCP*, Primary care provider.

UC providers reported continuing education, urgent dermatology clinics, and teledermatology as strategies to improve dermatology access. Preferred educational formats included lecture series (71.0%) and self-study modules (71.0%). Most respondents (90.3%) agreed there should be greater access to dermatologists for urgent skin concerns, and nearly all (96.8%) supported urgent dermatology clinics. While synchronous teledermatology was preferred over asynchronous (71.0% vs 29.0%), 67.7% were open to using asynchronous consults. If teledermatology services were available, 64.5% said they would be less likely to refer patients for in-person consultation.

## Discussion

Our findings highlight factors that influence patient referrals from UC to dermatology and UC providers’ experiences managing urgent dermatologic conditions. These insights may inform improvements in referral triage, referral completion, and dermatology support within UC settings. Previous studies, mostly in primary care, identified that both patient-related and provider-related factors impact referral decisions.^5^, ^6^, ^7^, ^8^, ^9^, ^10^, ^11^, ^12^, ^13^ In our study, several patient-related factors, including race, ethnicity, insurance, UC location, diagnosis, and PCP status, predicted referrals from UC to dermatology. Non-White patients had higher odds of referral, possibly reflecting greater disease severity at presentation or reduced physician confidence in managing disease in skin of color. Studies on atopic dermatitis, hidradenitis suppurativa, and psoriasis have reported greater disease severity in African American patients.^18^, ^19^, ^20^ Longstanding inadequacies in skin of color representation in medical education resources are demonstrated by increased diagnostic accuracy and confidence in medical students assessing skin conditions on fair skin compared to skin of color.^21^^,^^22^ Our survey supports lack of provider confidence as a factor, with 60.0% of UC providers reporting confidence in dermatologic care only some of the time.

While Forrest et al^5^ noted insured patients had higher referral odds from primary care, our study found uninsured UC patients were more likely to be referred. A possible explanation is that uninsured patients may lack a PCP for further follow-up, prompting UC providers to instead refer directly to dermatology. Although lack of PCP was not listed as a referral reason in our survey, the 1 respondent who chose “other” indicated “no primary care for follow-up” as a reason for referring. Like Forrest et al, our study showed Medicare patients had lower odds of referral, which may reflect greater continuity of care or existing relationships with PCPs within this population. Referral likelihood also varied by location. More distant UC sites referred patients less frequently, aligning with prior observations that rural physicians tend to refer less often.^23^

Diagnosis significantly influenced referrals. Conditions that often require procedural intervention (eg, viral wart, skin lesion, or cyst) and chronic conditions (eg, hidradenitis suppurativa) had higher referral rates, in contrast to acute conditions (eg, skin infections, contact dermatitis). Our survey supports this finding, with 74.2% of providers listing specialized procedures among the top reasons for referrals and 67.7% listing chronic conditions needing specialized care. UC providers rarely referred based solely on patient request.

The UC survey also explored clinical uncertainty as a factor affecting referrals. Diagnostic uncertainty (58.1%) and treatment uncertainty (22.6%) were among the top 3 reasons for referral. This aligns with reported findings that greater stress from uncertainty leads to increased referrals.^10^ Given the frequency of dermatologic presentations in UC and frequent UC provider uncertainty, additional education could be helpful. UC providers favored didactic formats (lectures and self-study modules) over hands-on clinical shadowing. Since hands-on methods have been shown to more effectively drive behavioral change, balancing educational impact and provider preferences is important.^24^

Most UC clinicians were open to teledermatology, preferring synchronous over asynchronous consultations. Implementing either modality may improve triage of urgent dermatologic concerns.^25^^,^^26^ Teledermatology performs comparably to in-person consultation in UC settings and may eliminate the need for in-person visits.^15^^,^^27^ Referral completion rates are higher with a teledermatology triage model, which is important given the low 47.9% referral completion rate in this study.^15^ Nearly all surveyed providers supported establishing urgent-access dermatology clinics to enable same-day appointments and reduce wait times. Although successful at several institutions, barriers such as space, staffing, and finances have limited widespread implementation.^16^^,^^28^, ^29^, ^30^

## Limitations

Study limitations include limited generalizability from a single health system, potential self-selection and response bias in the clinician survey, unmeasured provider characteristics (eg, training background, years of experience), no assessment of skin disease severity in UC, and lower survey response rate. To mitigate survey bias, the survey was anonymous, brief, neutrally worded, and distributed to all UR UC clinicians. Although this quantitative retrospective cohort analysis and survey study provides additional insight on dermatologic care in UC, future qualitative studies (ie, interviews, focus groups, open-ended surveys) could provide additional context for referral decision-making and improved dermatologic support in UC settings.

## Conclusion

This study identified key patient and provider factors influencing dermatology referrals from UC, including race, ethnicity, insurance status, clinic location, diagnosis, PCP, need for procedures, and clinician uncertainty. Despite a high burden of patients presenting to UC with acute dermatologic concerns, only a small proportion were referred, and less than half of those referred completed their dermatology appointments. Our findings highlight opportunities to improve access to dermatologic care through targeted UC provider education, teledermatology implementation, and establishment of urgent-access dermatology clinics. These strategies may enhance referral appropriateness, reduce delays in specialty care, and improve management for patients with urgent skin conditions.

## Conflicts of interest

Dr Richardson has served as an investigator for AstraZeneca and Incyte and as a consultant for Merck and Immunovant. Amanda Rodriguez Orengo, Dr Moynihan, Dr Green, and Dr Ryan Wolf have no conflicts of interest to declare.
